# Risks of outbreaks: The health concerns of internally displaced persons in Transcarpathia, Ukraine

**DOI:** 10.1016/j.nmni.2023.101106

**Published:** 2023-02-18

**Authors:** Pavlo Petakh, Aleksandr Kamyshnyi

**Affiliations:** Department of Microbiology, Virology, and Immunology, I. Horbachevsky Ternopil National Medical University, 46001, Ternopil, Ukraine; Department of Biochemistry and Pharmacology, Uzhhorod National University, 88000, Uzhhorod, Ukraine; Department of Microbiology, Virology, and Immunology, I. Horbachevsky Ternopil National Medical University, 46001, Ternopil, Ukraine

**Keywords:** Displacement, Infectious diseases, Vaccination, Cross-border cooperation, Migration

Dear Editor,

The invasion of Ukraine by Russia has resulted in widespread displacement, injury, and death, affecting approximately 6.2 million people or 15% of the country's total population [1]. The Transcarpathian region, located in the South-West of Ukraine, has received over 350,000 internally displaced persons (IDPs) since the start of the invasion. This remote region of Ukraine, which borders four EU countries and has a population of 1.3 million, has become a major destination for IDPs, with some of them moving on to bordering EU countries and being recognized as refugees.

The long-term health of IDPs is of utmost concern, especially those who are housed in temporary facilities for extended periods [2]. These facilities are often overcrowded, under-resourced, and located in remote areas, making the IDPs housed there highly vulnerable to communicable diseases. The IDPs in these facilities are often those with the fewest resources, putting them at risk of outbreaks that could potentially spread to neighboring EU countries.

The risk of communicable diseases in the Transcarpathian region predates the conflict. In late 2021, a case of acute flaccid paralysis caused by cVDPV2 was recorded, along with several cases in asymptomatic contacts [3]. The coverage of vital vaccination programs has been severely impacted by the invasion, making it difficult to locate and vaccinate children who have been displaced both within and outside the country. Many of these children do not have documentation, so their vaccination status is often unknown, and there is low awareness of the importance of vaccines among some hard-to-reach communities, particularly in rural areas that have recently been flooded with IDPs. The coverage of the catch-up inactivated polio vaccine campaign in the Transcarpathian region was only 35% as of March 16, 2022. The risk of an increase in measles cases is also a concern due to insufficient coverage in childhood immunization (Ukraine experienced outbreaks of measles before the war, most recently from 2017 to 2019) [4]. The background rate of meningococcal infection in the Transcarpathian region is also a major concern, with the incidence from 2016 to 2018 exceeding average national indicators by several times [5].

The EU border countries have recognized the complexity of the situation and are acting to mitigate the risks of outbreaks. A cross-border cooperation program with Slovakia, Hungary, and Romania has been established and has funded the construction of a microbiological laboratory at the regional infectious disease hospital, which will speed up the diagnosis of infectious diseases. Additionally, UNICEF has financed educational efforts on IDP vaccination. Dr. Pavlo Kolesnyk, in partnership with international organizations, has opened a clinic where IDPs can receive vaccinations.

The migration and humanitarian crisis in Transcarpathia poses a severe risk of outbreaks of infectious diseases not only for Ukraine but also for bordering countries. It is crucial that international partners and influential organizations continue to support Ukrainian healthcare authorities in addressing the critical issue of vaccination.

## Contributions

P.P. conceptualized the idea, reviewed the literature, and prepared the first draft of the letter. A.K. reviewed the letter. Both authors have read and approved the final letter.

## Funding

No external funding was used in the preparation of this letter.

## Transparency declaration

The authors declared no potential conflicts of interest concerning the research, authorship, and/or publication of this article.
